# Contradictory Reasoning Network: An EEG and fMRI Study

**DOI:** 10.1371/journal.pone.0092835

**Published:** 2014-03-25

**Authors:** Camillo Porcaro, Maria Teresa Medaglia, Ngoc Jade Thai, Stefano Seri, Pia Rotshtein, Franca Tecchio

**Affiliations:** 1 LET’S-ISTC-CNR, Rome, Italy; 2 Phylosophy Department, Roma Tre University, Rome, Italy; 3 Aston Brain Centre, School of Life and Health Sciences, Aston University, Birmingham, United Kingdom; 4 Behavioural Brain Sciences Centre, School of Psychology, University of Birmingham, Birmingham, United Kingdom; 5 Department of Neuroimaging, IRCCS San Raffaele Pisana, Rome, Italy; George Mason University, United States of America

## Abstract

Contradiction is a cornerstone of human rationality, essential for everyday life and communication. We investigated electroencephalographic (EEG) and functional magnetic resonance imaging (fMRI) in separate recording sessions during contradictory judgments, using a logical structure based on categorical propositions of the Aristotelian Square of Opposition (ASoO). The use of ASoO propositions, while controlling for potential linguistic or semantic confounds, enabled us to observe the spatial temporal unfolding of this contradictory reasoning. The processing started with the inversion of the logical operators corresponding to right middle frontal gyrus (rMFG-BA11) activation, followed by identification of contradictory statement associated with in the right inferior frontal gyrus (rIFG-BA47) activation. Right medial frontal gyrus (rMeFG, BA10) and anterior cingulate cortex (ACC, BA32) contributed to the later stages of process. We observed a correlation between the delayed latency of rBA11 response and the reaction time delay during inductive vs. deductive reasoning. This supports the notion that rBA11 is crucial for manipulating the logical operators. Slower processing time and stronger brain responses for inductive logic suggested that examples are easier to process than general principles and are more likely to simplify communication.

## Introduction

The ability to reason, crucial for effective social interactions and for the solution of common practical problems, is among the most advanced human intellectual abilities. Contradiction, as a sub-class of reasoning process is one of the cornerstones of human rational reasoning and is part of everyday life and communication. In debates or conversations, we usually examine a statement and evaluate the validity of its content before agreeing with or arguing against what is being said. In western culture, the elements normally used in a conversation derive from a framework based on the Aristotelian Square of Opposition (ASoO). Formally, the ASoO is based on categorical statements, either universal statements related to the totality, expressed by the logical operator (quantifier) *All* or particular statements related to a small subset of it, expressed by logical operator *Some*. When contradicting a generic statement, we tend to search for counter examples. For instance, to refute the statement ‘All swans are white’, we might argue that ‘The Australian swan is black’. In logic, the first statement (i.e. ‘*All*…’) is contradicted by the formulation ‘Some swans are black’ (i.e. ‘*Some*…’). Categorical propositions may be used in various logical relationships; in particular a premise-conclusion pair can be contradictory or non-contradictory [1]. In the ASoO the contradiction between a first categorical proposition (premise) with a second categorical proposition (conclusion) can be achieved by inverting the logical operators *All* with *Some* and the attribute, i.e. ‘white’ with ‘black’ in the example above. Two main types of logical reasoning can be distinguished: inductive when we derive a rule based on a series of observations, and deductive when we formulate an example based on a rule. When the premise is a particular example (‘*Some’*) and the conclusion is a universal rule (‘*All’*), their relation is assessed using inductive reasoning. In contrast deductive reasoning is needed to assess the reverse relation: how a universal rule (‘*All’*) applies to a particular example (*‘Some’*).

In the past decade, studies on conditional reasoning [2] and syllogism [3]–[9] have contributed to shed light on the neural basis of logical reasoning. Using such logical structures, these studies have consistently reported activation of distributed cortical and sub-cortical networks, including those associated with language processing and semantic and visuo-spatial skills (see for review [10]–[15]). Lateralized effects have also been reported, with the left hemisphere playing a key role in linguistic processes and the right hemisphere mainly supporting logical reasoning [11], [12], [16], [17], although other studies have found key linguistic and logical processes to be in different sub-areas in the left hemisphere [5], [7], [8].

To our knowledge, the neural correlates associated with different components of contradictory thinking, a simple but fundamental logical process, are still largely unexplored. To this aim we assessed responses in the reasoning networks to logical structures devoted to the identification of contradiction.

Based on a protocol used in our previous high-resolution EEG study [18], we undertook a separate event-related fMRI experiment and attempted to integrate EEG and fMRI data. The purpose of the present study was to investigate the brain recruitment and specific timing during the identification of contradiction. We used a protocol based on ASoO premise-conclusion pairs, since it allowed deconvolving the neuronal responses reflecting contradiction identification and manipulation of the two logical operators while controlling for linguistic and semantic features of the sentences. To delineate processing related to the contradictory judgment, we contrasted contradictory (C, e.g. ‘All swan are white – Some swan are black’) to non-contradictory (nC, e.g ‘All swan are white’ – ‘Some swan are white’) premise-conclusion pairs. Furthermore, contrasting All-Some (AS: deduction, ‘All swan are white – Some swan are black’) with Some-All ( SA: induction, ‘Some swan are white – All swan are black’) sentence pairs, we manipulated the order of universal/particular logical operators in order to verify if inductive and deductive reasoning are subtended by different neural structures. We hypothesized that in this later case the order in which the two sentences (AS vs. SA) are presented may affect the way in which they are logically processed and that brain imaging could identify the neural correlates of the logical inversion operation, which is orthogonal to the content inversion operation.

Data of the experiment is available from the corresponding author upon request.

## Materials and Methods

### Participants

#### EEG study

data for the EEG study was collected during a previous study (reported in [18]) from eleven healthy native Italian speakers (mean age: 30.5 years; range: 24–38 years; six women).

#### fMRI study

Thirteen healthy native Italian speakers (mean age 31.1 range 25 – 42 years; 6 women) were recruited. Eight of these participants had also participated the EEG study.

None of the participants had a formal background in logic and none had prior history of neurological or psychiatric conditions. All participants had normal or corrected-to-normal vision. Participants gave their informed written consent after the nature of the study was explained to them. The study was approved by the Ethics Committee of Aston University, where the study was conducted.

### Stimuli and procedure

Stimuli were presented visually via a Dell PC using a paradigm developed on the E-Prime programming software (Psychology Software Tools, Inc.; http://www.pstnet.com). A 2×2 factorial design was used with the following factors: *Logical Operator* (Induction: Some-All, Deduction: All-Some) and *Contradiction* (Contradictory, Non Contradictory). Sentences were in Italian (the native language of the participants). The protocol consisted of 140 sentence pairs, equally represented in the following four forms: 35 *All… Some*… – Contradictory (AS-C), 35 *Some*… *All*… – Contradictory (SA-C), 35 *All*… *Some*… – Non-contradictory (AS-nC) and 35 *Some*… *All*… – Non-contradictory (SA-nC). The English translation of 4 typical sentences is provided in Table 1. In the AS-C and AS-nC the premise is universal (A: *All*) and the conclusion is particular (S: *Some*), whilst the conclusion is contradictory for the first set (AS-C) and non-contradictory for the other (AS-nC). Similar definitions apply for the SA-C and SA-nC. The sentences were identical as far as the logical operator manipulation; the only difference was the order in which the logical operators were presented, eliciting inductive or deductive reasoning. The C and nC sentences had identical grammatical structure and were matched as much as possible for length and complexity.

**Table 1:** Stimuli Example.

	Universal – Particular	Particular – Universal
**Contradictory**	All swans are white - Some swans are black	Some swans are white - All swans are black
	[**AS-C**]	[**SA-C**]
**Non-contradictory**	All swans are white - Some swans are white	Some swans are black - All swans are black
	[**AS-nC**]	[**SA-nC**]

An example of the four experimental conditions. The same number of premise-conclusion pairs was presented for each condition. Acronyms are indicated in square brackets.

**Figure 1:**
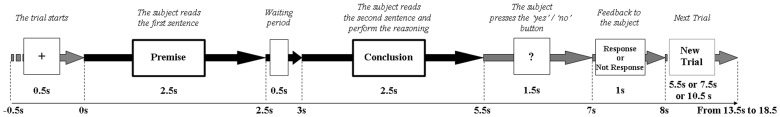
Experiment setup. The beginning of the trial was signalled by a “+” appearing in the centre of screen for 0.5 s, followed by the presentation of the premise categorical proposition for 2.5 s. Thereafter the sentence disappeared for 0.5 s (Waiting period), followed by the presentation of the conclusion categorical proposition for 2.5 s. Then, a question mark appeared for 1.5 s, requiring the subject to press the yes/no button in the case the contradiction was estimated correct/not correct independently of the sentence content. His/her response was accepted only within this period. Finally, a feedback advised the subject whether any button had been pressed or not, but no indication about the correctness of the contradiction identification. The ‘New Trial’ appearing on the screen (5.5 or 7.5 or 10.5 s randomly chosen) advised the subject that a next trial was starting.

Prior to the experiment, participants received a training session on a separate set of stimuli to help them to familiarize with the task and the recording environment. Participants (Ps) were asked to evaluate whether the conclusion was contradictory or not with respect to the premise, independent of the correctness of the sentence content. The trial sequence started with 0.5s fixation to alert the Ps of the beginning of the trial, the premise sentence was then presented for 2.5s followed by a 0.5s fixation and the conclusion sentence for 2.5s (Figure 1). The trial ended with the presentation of a question mark for 1.5s, during which participants were asked to perform a button press (right hand = contradictory, left hand = non-contradictory). Participants were instructed to refrain from responding if unable to make a clear choice. Three randomly selected inter trial intervals (5.5 or 7.5 or 10.5s) were used for the fMRI experiment, to facilitate the estimation of the Hemodynamic Response Function (HRF) in the MRI experiment [19]. A fixed 1s inter trial interval was used for the EEG procedure. Sentence pairs were presented in a random order.

### Analysis of behavioral data

Repeated measures analyses of variance (ANOVAs) were separately applied to the percentage of errors and the reaction times of correct responses (dependent variables) with *Logical Operator* (Induction [SA], Deduction [AS]) and *Contradiction* (Contradictory [C], Non-contradictory [nC]) as within-subject factors using SPSS15 (SPSS Inc, Chicago IL, USA). Threshold for significance was set at p<0.05.

### EEG data acquisition

Scalp EEG signals were recorded continuously during the protocol using an EGI data acquisition system (Electrical Geodesics, Eugene, OR, USA; http://www.egi.com) with the third generation of EGI dense array net, the HydroCel Geodesic Sensor Net VR (128-channel HCGSN [20]). Gain and zero calibration were performed before the start of each EEG recording; channel impedances were kept below 50 kΩ for all net sensors [21]. Amplified EEG signals were sampled at 500 Hz (anti-aliasing filter at 200 Hz) and stored for off-line processing. All channels were referenced to the vertex (Cz) electrode. Source analysis of this dataset was presented in [18].

### EEG data analysis

A semiautomatic Independent Component Analysis (ICA) procedure was applied to characterize artifactual non-cerebral activities, i.e. eye movements, muscular artifacts and environmental noise, and remove them without rejecting the contaminated epochs [22]–[24]. EEG analysis was then performed after back-projecting all the non-artifactual ICs in the original signal space. To identify specific neural regions involved in different phases of the task, the “cleaned” data were then averaged, triggered on the presentation of the first sentence. A 7-second time window that comprised the whole trial (premise 2.5 s + blank screen 0.5 s + conclusion 2.5 s + response 1.5 s) was chosen, and reasoning-associated Global Field Power (GFP, [25]) was computed. We assessed brain activation patterns performing the same contrasts for EEG and fMRI. The ‘C vs. nC’ and ‘SA vs. AS’ difference Event Related Potential (ERP) was subject to source analysis using sLORETA algorithm [26] as implemented in CURRY 6 (Neuroscan, Hamburg, Germany, http://www.neuroscan.com/). sLORETA was computed using a regular grid with a spacing of 4 mm throughout the brain region and a four shell spherical head model. The results were projected onto the template brain of the Montreal Neurological Institute (MNI). Anatomical sources of the ERPs were identified blindly to the results of the fMRI data.

The current analysis extended the analysis presented in the previous study on the same data set [18] now integrating the dynamics of specific involved structures.

### Statistical analysis of EEG data

To identify which and when cerebral events were crucial for a specific processing step, corresponding ANOVA designs were performed on maximal channel ERPs including *Logical Operator* (Induction [SA], Deduction [AS]), *Contradiction* (Contradictory [C], Non-contradictory [nC]) 2-levels within-subjects factors and the n-level *Activation latency* (lat_1_,lat_2_,…, lat_n_) within-subjects factor. The selection of latencies lat_1_, lat_2_ … lat_n_ corresponding to ‘relevant events’ was performed implementing a data-driven procedure. We selected the two channels of maximal amplitude (positive and negative polarity at earliest latency) of the grand average ERP across the four conditions (SA, AS, C and nC). Noteworthy, the same two channels (F9 and F10) displayed the maximal amplitudes all along the task and also in the four conditions separately (AS, SA, C, nC). ‘Relevant’ latencies lat_1_, lat_2_ … lat_n_ were those which the t-test analysis calculated millisecond by millisecond indicated as differing for at least one of two maximal channels ERPs in in either conditions’ comparison (Induction vs. Deduction or Contradictory vs. Non-contradictory).

Threshold for significance was set at p<0.05 and trends were reported for p<0.10. Effects were reported as results only when below significance. Post-hoc comparisons for each latency point were reported for the channel with strongest effect.

### fMRI data acquisition

All data were acquired with a 3 Tesla Siemens Trio MRI scanner (Siemens Medical Systems). Functional images were acquired with an 8-channel birdcage phased-array head coil using a gradient echo sequence with the following parameters, TR = 3.0 s, TE = 30 ms, flip angle  =  90° Matrix 64×64, FOV 192 cm, 3×3 mm in-plane voxel resolution and 3 mm slice thickness. A total of 44 slices per full brain volume and between 765 to 800 volumes per participant was acquired in a single session.

### fMRI data analysis

The fMRI data were analyzed using SPM8 (Wellcome Department of Imaging Neuroscience, London; www.fil.ion.ucl.ac.uk/spm). Pre-processing of the data included slice time correction, spatial realignment to correct for movement artifacts and motion by distortions interactions and normalized to the MNI standard space. The data were re-sampled given a 2×2×2 voxel size and smoothed using 6 FWHM Gaussian Kernel to account for residual inter-subject differences and to accommodate assumptions of random field theory used for family wise error corrections [27].

We first estimated the effect size for each participant on each of the four conditions (recall 2×2 design) using the general linear model [28]. Each condition was modeled by a separate regressor; the onsets of each trial corresponded to the time when a correct response was given. In addition, response time and length of the sentences were included as covariates to control for potential stimulus and response confounds. Error and non-response trials were modeled as separate regressors. All the regressors were convolved with two bases functions: the canonical HRF [29] and its derivatives that capture fluctuations in response onset [30]. To correct for signal changes due to head movement, the 6 realignment parameters were included in the design matrix. An additional set of harmonic regressors was used to account for any temporal low-pass frequency variance within the data that is typical to fMRI signal with a cut-off of 1/128 Hz.

Consistent effects across participants (random-effects, second-level analysis [31]) were then tested using the general linear model framework. The data for the described model were the estimated effect size of the 4 experimental conditions for each subject (AS-C, AS-nC, SA-C, SA-nC). Plots of the averaged estimated HRF response size represent the responses (the first Eigen-variant) of a 3 mm^3^ sphere centered on the maxima group response. We used a mixed cluster and peak threshold [32]. We report clusters with a peak at P<0.001 uncorrected and at least 75 contiguous voxels showing Z > 2.58, unless specified otherwise. To cross validate the reliability of our observations we tested for overlap between the EEG sources and the regions of increased BOLD signal in the fMRI experiment. This was done by using a 5 mm sphere around the peak of the sources observed from the independent EEG study and analysis (see above section) for small volume correction to the fMRI regions.

## Results

### Behavioral performance during the fMRI and the EEG experiments

Results of behavioral performance are summarized in Table 2. Participants performed Particular - Universal (SA: Induction) and Universal - Particular (AS: Deduction) trials with comparable accuracy (> 95%), both for the contradictory and non-contradictory conditions (p > 0.150). Such low error rate suggests that logical inference based on the Aristotelian categorical proposition structure is a natural process that is easily performed by all participants, even without ever having had any formal training in logic.

**Table 2:** Behavioral data.

	EEG		fMRI	
	Universal – Particular	Particular – Universal	Universal – Particular	Particular – Universal
	Correct responses (%)			
**Contradictory**	97.5±1.9	98.0±3.2	97.3±2.4	96.2±5.9
**Non-contradictory**	98.5±1.7	97.9±2.2	95.1±1.9	97.3±2.4
	**Reaction Times (ms)**			
**Contradictory**	490±105	550±117	512±107	561±96
**Non-contradictory**	501±101	513±107	505±135	539±161

In each of the four experimental conditions, for EEG and fMRI data, mean across participants (± standard deviation, SD) of the percentage of correct responses and of the reaction times, i.e. the time between the question mark appearance and the subject response (see Fig.1).

Reaction Times showed a strong *Logical Operator* effect [F(1, 12) = 14.555, p<0.005], with longer reaction times seen for inductive reasoning, when a Universal rule had to be inferred based on an example. No main effect for *Contradiction* or an interaction between Logical Operator*Contradiction were observed [p>0.5 for both]. Observed longer RT for SA than AS (*Logical Operator* effect p<0.005) but no RT difference between C and nC (*Contradiction* effect p>0.5) in both the EEG [18] and the fMRI experiments. To further test whether our participants developed the heuristic strategy in the course of the experiment (i.e. produced the identification based on the inversion of the attribute instead of considering the whole sentences), we divided the experiment into four parts and performed an ANOVA adding *Time* (1°, 2°, 3°, 4° period) as within-subjects factor to *Logical Operator* (AS, SA) and *Contradiction* (C, nC). The results revealed that participants improved their overall performance along the course of the experiment (*Time* effect F(3,36)  =  15, p<0.001) and were faster to compute AS than SA logical operation (F(3,36)  =  9.83, p =  0.011) with no effect of *Contradiction*. Importantly, *Time* did not interact with either of the manipulations (p > 0.200 for both Time*Contradiction and Time*Logical Operator), indicating that participants did not change their strategy during the course of the experiment.

### Brain activation - EEG data

#### Dynamics of Logical Operator and Contradiction processing

**Figure 2:**
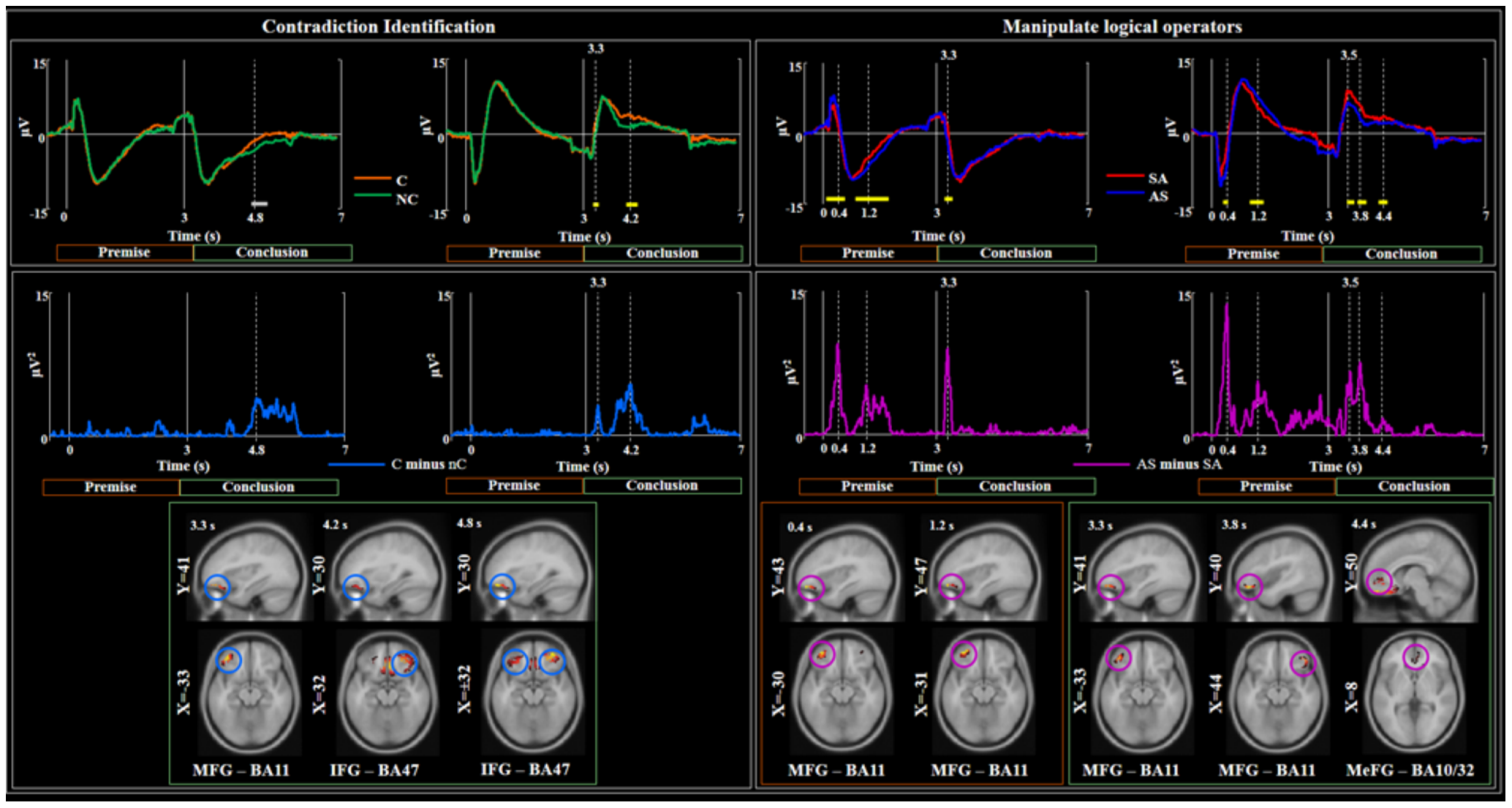
Neural structures to identify contradiction and manipulate logical operators –EEG data. **Top row** - Grand average Event Related Potential (ERP) of the two channels of maximal amplitude (positive and negative polarities at the first peak) compared between contradictory (C) and non-contradictory (nC) (on the left, orange and green lines respectively) and *All-Some* (AS) and *Some-All* (SA) (on the right, red and blue lines respectively) conditions. In accordance with figure 1, time t = 0 s refers to premise presentation (full vertical line, orange box) and the conclusion is displayed 3 s later (full vertical line, green box). Horizontal yellow (grey) segments indicate time periods when the difference between conditions where significant at paired 2-tails t-test p<0.05 (p<0.1). **Middle row** - Grand average differences of the two channel powers between contradictory (C) and non-contradictory (nC) (on the left, blue lines) and *All-Some* (AS) and *Some-All* (SA) (on the right, magenta lines) conditions. **Bottom row** - Localization results at latencies of GFP peaks (vertical dashed lines in the GFP traces) of corresponding contrasts are expressed in MNI template brain. GFP was found for the C vs. nC contrast only in the conclusion (green box), while cerebral recruitment in correspondence to premise (orange box) and conclusion processing (green box) were evidenced for SA vs. AS contrast.

When comparing the two channels of maximal amplitude (positive polarity at the first peak F9, negative polarity at F10), ERP peaks showed significant differences (p<.05, Figure 2 top row) at 400, 1200, 3300, 3500, 3800, 4400 ms in the Induction vs. Deduction contrast, and at 3300, 4200, 4800 ms in the Contradictory vs. Non-contradictory contrast. Based on this finding, these 8 latencies were included in the ANOVA design.

The full ANOVA design including the 3 within-subjects factors, showed a significant Logical Operator*Contradiction*Activation latency interaction factor [F(7,28) = 3.080, p = .016] revealing a different behavior at different latencies when manipulating the logical operator or recognizing the contradiction. Consequently, for each latency reduced models were analyzed, and latencies were considered of interest whenever at least one factor (*Logical Operator* or *Contradiction*) was significant. As detailed in Table 3 a trend for Logical Operator*Contradiction was found at 400 and 3300 ms. *Logical Operator* factor reached statistical significance at 400, 1200, 3300, 3500, 3800 and 4400 ms and *Contradiction* factor was significant at 3300, 4200 ms and showed a trend at 4800 ms.

**Table 3:** Statistics of neural structures activation dynamics.

Latency (ms)	Effect	F(1,10)	p
400	(Logical Operator * Contradiction)	3.583	**.**091
	Logical Operator	12.571	**.**005
1200	Logical Operator	20.840	.001
3300	(Logical Operator * Contradiction)	3.322	.113
	Logical Operator	10.158	.015
	Contradiction	7.545	.020
3500	Logical Operator	9.696	.012
3800	Logical Operator	14.792	.003
4200	Contradiction	5.160	.036
4400	Logical Operator	4.784	.046
4800	Contradiction	3.472	.094

Results of reduced model ANOVA designs for each latencies.

### Structures crucial for identification of contradiction

As expected (since all premises are presented in the two contrasting conditions) the analysis of ERP data contrasting C vs. nC premise-conclusion pairs revealed no differences in GFP values between 0 and 3000 ms during the whole premise processing (Figure 2). The first GFP peak was seen 300 ms after the onset of the conclusive sentence. The cerebral activation at that time point localized to the left MFG (BA 11 - Figure 2, Table 4). The activity at the next GFP peak, occurring around 1.2 s after the beginning of the trial, was localized in the right IFG and corresponded to processing of the conclusion.

**Table 4:** Neural structures to identify contradiction and manipulate logical operators – EEG data.

		BA	X	Y	Z	Latency	
			(mm)	(mm)	(mm)	(s)	
**Contradictory vs. Non-Contradictory**						Prem	Conc
**Frontal Lobe**	Middle Frontal Gyrus (L)	**11**	–33	41	–14	3.3	**0.3**
	Inferior Frontal Gyrus (R)	**47**	32	30	–17	4.2	1.2
**Induction: Particular – Universal (SA) vs. Deduction: Universal – Particular (AS)**						Prem	Conc
**Frontal Lobe**	Middle Frontal Gyrus (L)	***11***	–*30*	*43*	–*15*	***0.4***	
		***11***	–*31*	*47*	–*15*	*1.2*	
		**11**	–33	41	–14	3.3	**0.3**
	Middle Frontal Gyrus (R)	**11**	44	40	–14	3.8	0.8
	Medial Frontal Gyrus/ACC (R)	**10/32**	8	50	0	4.4	1.4

Localization results in MNI coordinate and latencies of GFP peaks of contrasted EEG data. Latencies are expressed relative to both the premise presentation (t = 0, first latency column, Prem) and the conclusion presentation (t = 0 at 3s after premise presentation, second latency column, Conc); this also means that latencies <3s of the first latency column refer to premise processing and latencies > 3s relate to conclusion processing. Latencies in bold indicate reasoning steps occurring in the same processing phase after premise and conclusion presentation. Dynamics of lateralization of brain recruitments is underlined by dedicated boxes for left (L) and right (R) activated areas. In italics are indicated cerebral processing of the premise.

### Logical operator processing structures

ERPs obtained by contrasting the activity in response to SA vs. AS premise-conclusion pairs showed a first GFP around 0.4 s after premise presentation, with the largest activation in the left middle frontal gyrus (MFG-BA11) (Figure 2, Table 4). This area was also active during the subsequent GFP peak around 1.2 s following the onset of the premise. The first peak after the onset of the conclusion, occurred around 300 ms, at a similar latency as the earliest peak after premise presentation and was localized in the same region (left MFG-BA11, Table 4), plausibly reflecting the similar processing step. The processing of the conclusion was characterized by shorter latency and weaker amplitude than that of the premise (Figure 2). We note that this early (0.3 s post conclusion onset) GFP peak observed in the left BA11 was sensitive to both the contradiction and the logical operator manipulations. The next activity peak during processing of the conclusion was generated in the right MFG (BA 11, Table 4) around 800 ms after the onset of the second sentence presentation. The activity spread to the right hemisphere MeFG (BA 10) and ACC (BA 32) where it peaked 600 ms later, at 1.4 s (Figure 2, Table 4).

A dynamic interactive process emerged from the onset of the conclusion is the process of contradictory reasoning. An early response at 0.3 s in the left MFG (BA 11) potentially involved in comprehension process signals the recognition that the two sentences have different words. We infer that this early peak in the left MFG is not yet part of the contradictory reasoning process but reflects comprehension processes since these effects are evident also for the premise sentence when ‘*All*’ and ‘*Some*’ are contrasted. The inversion of the logical operators that followed this stage leading to inductive/deductive reasoning and involved the right MFG (BA 11) at 0.8 s. This was then followed by activation of the right IFG at 1.2 s during the comparison of semantic content of the conclusion with respect to the premise. Finally, re-evaluation of the logical operators was associated with the right MeFG activation at 1.4 s. It is interesting to note that within the reasoning time-window, inversion of the logical operators (0.8 s at right BA11) preceded the semantic comparison of the sentence content (1.4 s, at the right BA47).

The EEG-derived activation data showed that the right hemisphere contributed more than the left during the whole reasoning time window (0.8 – 1.4 s from the presentation of the conclusion) for both the identification of the contradiction (C vs. nC, Figure 2 left, Table 4) and the logical operator manipulation (SA vs. AS, Figure 2 right, Table 4). A left-hemisphere involvement was present earlier 0.3 s in all contrasts (Figure 2 left).

### Brain activations - fMRI data

#### Overall task relevant activation

**Figure 3:**
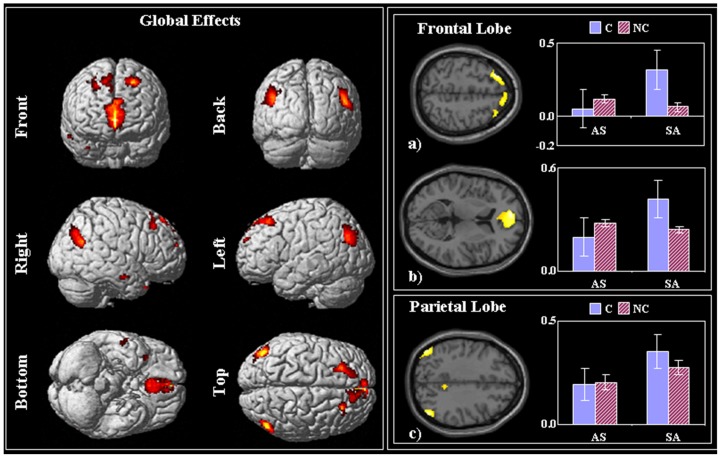
Overall task relevant activations – fMRI data. On the left, the SPM-T (P<0.05 FWE-corrected) of the global task effect (stimuli vs. fixation) is overlaid on a template rendered brain. On the right, the SPM-T is overlaid on MNI template T1 axial slices. The plots represented the effects size in regions of interest (y-axis fMRI effect size in arbitrary units). a) Right superior frontal gyrus, BA 8 [MNI: –26 32 54]; b) medial frontal cortex, BA10, (MNI: –6 54 8), c) left inferior parietal lobule, angular gyrus, BA 39 [MNI: –48 –76 36]. Indices: C, contradictory; nC, non-contradictory; AS, *All*-*Some*; SA, *Some*-*All.*

In line with previous fMRI studies, we first tested for the main effect of reasoning, independent of the specific condition. i.e. cerebral responses across all the four conditions when compared to the fixation baseline. Note that this general contrast includes regions involved in processing of visual stimuli, reading, comprehension, decision, preparation of motor response, regions that are involved in non-specific but task relevant cognitive demands as well as regions directly performing the reasoning task. Given the non-specificity of this contrast, we report only results that survived whole brain family-wise error correction (FWE) at the cluster level p<0.05. Overall, regions within the bilateral prefrontal, parietal and left temporal cortices showed increased activations during task versus baseline (Table 5; Figure 3). Interestingly, while performing the task the early sensory structures (visual cortex and thalamus) showed a decrease in activity relative to baseline (Table 5). This suggests that while judging the logical reasoning, participants were engaged in internal mental processing while attempting to ‘block’ external distractions [33].

**Table 5:** Overall task relevant activations – fMRI data.

	Increased Activity - Location	BA	X	Y	Z	z-value
			(mm)	(mm)	(mm)	
**Frontal Lobe**	Medial Frontal Gyrus (L)	**10**	–6	54	8	4.77
	Superior Frontal Gyrus (L)	**6/8**	–26	32	54	4.67
	Medial Frontal Gyrus (R)	**10**	2	54	–2	4.95
	Superior Frontal Gyrus (R)	**6/8**	36	32	54	3.86
**Parietal Lobe**	Precuneus (L)	**31**	–10	–54	30	3.71
	Angular Gyrus (L)	**39/40**	–48	–76	36	4.52
	Angular Gyrus (R)	**39/40**	48	–74	36	4.46
**Temporal Lobe**	Middle Temporal Gyrus (R)	**21**	52	–64	24	4.28
**Limbic Lobe**	Anterior Cingulate (L)	**24/ 32**	–2	36	0	4.51
	Posterior Cingulate (R)	**31**	10	–52	22	3.63
	**Decreased Activity - Location**	**BA**	**X**	**Y**	**Z**	**z-value**
			**(mm)**	**(mm)**	**(mm)**	
**Occipital Lobe**	Cuneus (L)	**18**	–8	–104	6	5.63
	Cuneus (R)	**18**	16	–102	8	5.55
**Sub-lobar**	Thalamus (L)		14	–8	8	6.28
	Thalamus (R)		14	–4	6	6.23

fMRI results in MNI coordinates. See text.

### Structures crucial for identification of contradiction

**Figure 4:**
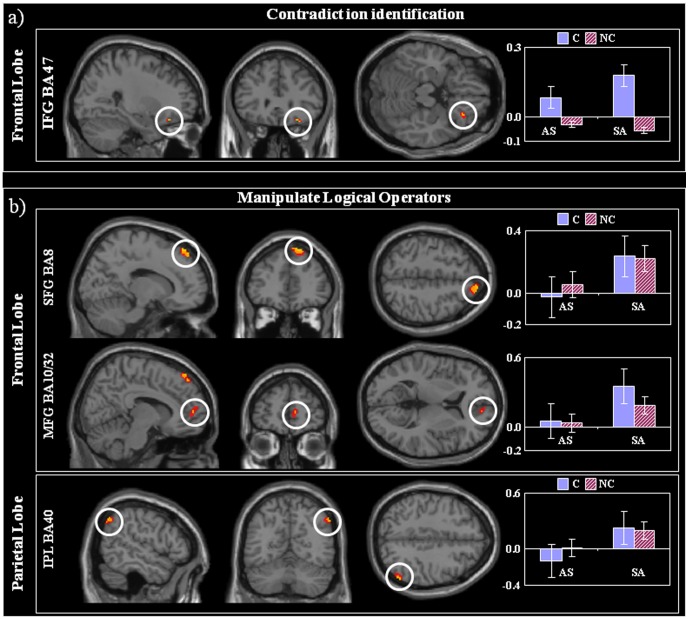
Neural structures to identify contradiction and manipulate logical operators – fMRI data. **a)** Contradiction Identification On the right, the SPM-T (orange, P<0.001; red P<0.005) of the contradictory effect (contradictory > non-contradictory) overlaid on MNI template T1. The plots represent the effects size in regions of interest (y-axis fMRI effect size in arbitrary units). Indices: C, contradictory; nC, non-contradictory; AS, *All*-*Some*; SA, *Some*-*All*. **b)** Manipulation of Logical Operators On the right, the SPM-T (orange, P<0.001; red P<0.005) of the logical operator effect (*Some*-*All* (SA) more than *All*-*Some* (AS)) overlaid on MNI template T1. The plots represent the effects size in regions of interest (y-axis fMRI effect size in arbitrary units).

The right inferior frontal gyrus (IFG, BA 47), and caudate nuclei showed increase activation during contradictory compared to non-contradictory trials (p<0.001, Figure 4a and Table 6). Within these regions there was no effect of the logical operator manipulation, suggesting that BA 47 responded in a similar way irrespectively to the manipulation of *All* or *Some*. Even though the size of these clusters was below our pre-specified threshold, the involvement of the right BA 47 and the caudate in decision-making, response selection and contradictory scenarios is consistent with previous literature (see discussion for further details). As expected, the fMRI responses of the right BA 47 overlapped with the contradictory EEG-source peak at 1.2 s. This overlap was again formally confirmed with small volume correction (SVC) using a 5 mm sphere centered at the EEG-source peak to the fMRI data. Using SVC, the response in right IFG was larger for contradictory statements than no-contradictory ones (FWE-corrected p = 0.008). The opposite contrast (nC> C) caused no activation above threshold.

**Table 6:** Neural structures to identify contradiction and manipulate logical operators – fMRI data.

Contradictory (C) > Non-contradictory (nC)		BA	X	Y	Z	z-value
			(mm)	(mm)	(mm)	
**Frontal Lobe**	Inferior Frontal Gyrus (R)^1^	**47**	26	30	–18	3.31
**Sub-Cortical**	Caudate (R)^1^		22	6	14	3.1
**Induction: Particular – Universal (SA) > Deduction: Universal – Particular (AS)**		**BA**	**X**	**Y**	**Z**	**z-value**
			**(mm)**	**(mm)**	**(mm)**	
**Frontal Lobe**	Medial Frontal Gyrus/ACC (R)	**10/32**	10	54	6	3.13
	Superior Frontal Gyrus (R)	**8**	14	42	54	3.83
**Parietal Lobe**	Inferior Parietal Lobule (R)	**40**	54	–62	44	3.25

fMRI results in MNI coordinates. See text. ^1^cluster size > 28 mm^3^.

### Logical operator processing structures

In line with the behavioral findings, we observed a greater BOLD signal change for inductive reasoning propositions ending with a Universal (SA) than for deductive reasoning - those ending with a Particular statement (AS) in the right superior frontal gyrus (SFG – BA 8), medial frontal gyrus (MeFG – BA 10) and the right inferior parietal lobule (IPL – BA 40, angular gyrus) (Figure 4b, Table 5). Within the above foci there was no reliable effects for the contradictory manipulation (p > 0.1), suggesting that responses to contradictory and non-contradictory sentences recruited these structures in a similar way. We note that the EEG activation peak (at 1.4s) in the right MeFG for the inversion of the logical operator overlapped with the fMRI activation at that region. This overlap was confirmed by applying SVC using a 5 mm sphere centered at the EEG peak to the fMRI data, the response in right MeFG was found to be FWE-corrected (p = 0.010). There was no above threshold activation for increased response to SA vs. AS.

### Relationship between behavior and brain activations

As detailed above, no effects of experimental conditions were observed on error rates. Reaction times were longer when contradiction identification was performed after a universal (condition SA) rather than a particular conclusion (condition AS). This finding lead us to investigate brain activation properties explaining the reaction difference in the two conditions (Reaction Time delay, RTd  =  RT(SA)-RT(AS)). In particular, we focused on the activity of brain regions emerging from the same SA vs. AS contrast. After checking variables distribution for gaussianity with Shapiro-Wilk statistics, a linear regression analysis was performed to identify possible cerebral activation features predicting the RTd.

EEG and fMRI studies indicated that specific areas were involved for manipulating the logical operator. We investigated if activation latency or current strength of right BA11 or BA10/32 (see Table 4) or BOLD intensity in BA10/32, BA8 and BA40 (Table 6) were able to explain the longer reaction time which occurred in SA vs. AS conditions (see Table 2). Investigating preliminarily the Pearson’s correlations with the reaction time delay (RTd), no association appeared with either EEG current strengths or fMRI BOLD. Instead right BA11 and BA10/32 activation latencies correlated with RTd. We used regression analysis to establish which of these displayed higher association. The regression analysis with RTd as dependent variable and right BA11 and BA10/32 activation latencies as independent variables (Stepwise method) indicated that only right BA11 activation latency (rBA11al) entered the model, as expressed by

**Figure 5:**
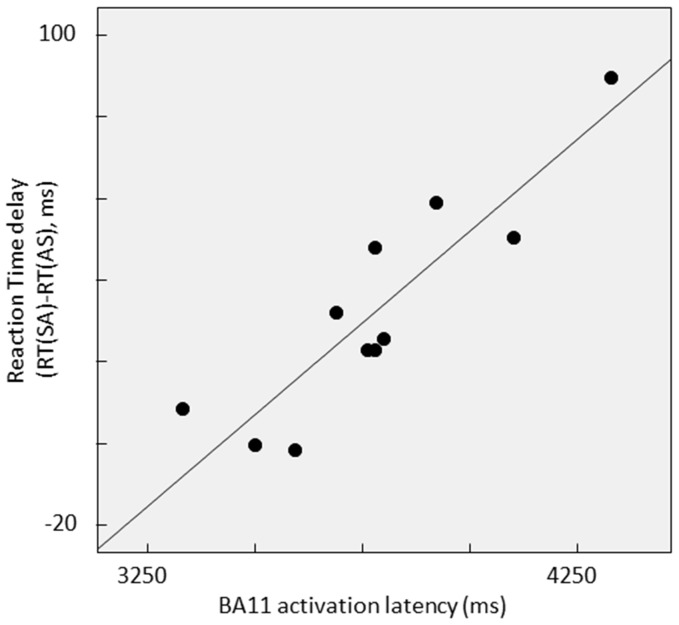
Relationship between behavior and brain activation. Scatterplot of individual data submitted to the linear regression analysis, with Reaction Time delay [RT(SA)-RT(AS)] as dependent variable and right BA11 activation latency as independent variable.

RTd =  –307.6 + 0.090 rBA11al (Figure 5).

The 80% of RTd variance was explained by this model [F(1,9) = 35,660, p<.001].

## Discussion

Going beyond our previous study using the Aristotelian Square of Oppositions [18], here we delineated the spatial temporal envelope of two processes that are keys to reasoning thinking: logical operators of deductive and inductive inferences and the detection of contradictions. We found that inductive (SA: Some-All ) reasoning was more difficult than deductive (AS: All-Some) reasoning and it was associated with increase responses of the right superior and medial prefrontal cortex (BA 8, 10, 32) and the inferior parietal (BA 40), in the fMRI. The EEG revealed more refined spatial-temporal properties of the reasoning processed. Here 0.8 s after the onset of the conclusion sentence the right MFG (BA 11) showed a different activity depending on the type of reasoning made. Interestingly the timing of right MFG (BA 11) peak predicted participants’ reaction time delay in inductive vs. deductive reasoning. The responses of the MeFG (BA 10) that were seen in the fMRI were observed later at 1.4 s. Participants found it equally easy to detect contradictory as well as non-contradictory sentences, however both the fMRI and EEG revealed that responses of the right MFG (BA 47) arising at 1.2 s after the onset of the conclusion sentence showed stronger responses to the contradictory than to non-contradictory sentences. Our data highlighted that specific spatial-temporal network properties subtend deduction/induction and identification of contradiction. We next discuss in more details each of the observed effects and potential reasons of some apparent inconsistencies between the fMRI and EEG data.

The limitation of our study is the relatively small simple size, since the statistics are prone to false negative as well as false positive results in this case. However, the reliability of the results emerges from convergent evidence provided by the two multimodal brain measurements (EEG and fMRI), the correlation of the behavioral reaction delays with the brain activation latencies, of the region crucial to perform the step differing between the two conditions, and the consistency of the findings with previous literature.

### Dynamics of reasoning network to identify an Aristotelian contradiction

The analysis of the EEG data enabled to describe the temporal sequence of the activation of structures within the prefrontal cortex necessary to solve an Aristotelian contradiction. In our previous EEG study [18] we focused on identifying the frontal network that was involved in all the four conditions. This revealed a bilateral temporo-polar cortices (BA 21, 38), bilateral Inferior Frontal Gyrus (IFG - BA 47) cortex, the Medial Frontal Gyrus (MeFG - BA 10, 11) and the Anterior Cingulate Cortex (ACC - BA 32) [18]. In this study we focused on identifying how the spatial layout of the network changes dynamically as a function of the reasoning conditions.

After the onset of the conclusion the left MFG was activated first (0.3 s) and at the almost same latency as following the onset of the premise (0.4 s). The involvement of the left MFG present in the premise and not just in the conclusion suggests a P300-like activation, reflecting semantic comprehension processing. This area is in fact part of the P300 generator structure [34].

Only after the onset of the conclusive sentence, a second response emerged about half a second after the initial P300 within the right frontal lobe structures that were associated with the logical operator manipulation (right MFG, 0.8 s after conclusion presentation, Figure 2). Four hundred ms later (1.2 s after conclusion presentation), contradiction identification involved the right hemispheric IFG whereas 200 ms later (1.4 s) ACC is activated.

While EEG-derived activation revealed the dynamics of the reasoning process, these structures were observed independently by both fMRI and EEG data, indicating a processing network with crucial contributions of distinct frontal lobe structures and a right prevalence in reasoning to identify an Aristotelian contradiction.

### Sub-system to manipulate the logical operators in contradiction identification

Our EEG and fMRI results provide evidence that specific neural structures are crucial for the manipulation of the logical operators while identifying the contradiction. The right inferior frontal cortex (rIFG - BA 47) is crucial for inferring contradiction based on the premise-conclusion semantic content. However, the logical operator processing (Induction and Deduction) mainly relies on the right medial frontal cortices (MeFG - BA 10/32, MFG - BA 11), regions accounting for depth of strategic reasoning [35]. EEG, fMRI and behavioral results all demonstrated that deductive reasoning when concluding with a particular statement is simpler and easier logical process than inductive reasoning when concluding with a universal statement. We hypothesize that the advantage of Deduction with respect to Induction - faster reaction time - depends on the fact that the former requires the analysis of a single example, whereas sentences terminating with *All* require exploring multiple examples or the absence of a counterexample.

A recent study based on a paradigm that required counterexamples to be used to refute invalid inferences showed activation in right prefrontal cortex [10].

A role of the right BA 47 and the caudate in prediction errors and surprise events was described in a study using words that had causal or non-causal relation to each other [36]. These authors report increased activation - amongst other regions - of the right BA 47 and caudate nuclei for the ‘surprise’ trials in which words were not causally related, compared to when they were. In light of these findings, we propose that increased responses to contradictory vs. non-contradictory trials may reflect an ‘intellectual surprise’ elicited by the conceptual violation originated by the contradiction.

The absence of reaction time differences between contradictory and non-contradictory pairs revealed similar cerebral processing load of the two premise-conclusion stimuli. Interestingly, longer reaction times were instead required when concluding with a universal statement. This delay was mainly due to longer activation time of the specific structure associated with manipulation of the logical operator during a universal statement. This brain-behavior relationship has the double implication of strengthening the reliability of the brain structures’ identification and of enabling a better comprehension of a relevant reasoning process. In fact, it emphasizes the efficacy of the present protocol, where by equaling all semantic and language-specific features of premise-conclusion pairs we were able to isolate the neuronal structures devoted to specific steps of the reasoning process.

Our results support the notion that specific examples more that general statements facilitate communication and teaching [37]. We showed that induction reasoning, 1) took longer to make, 2) were associated with increased fMRI/EEG responses in prefrontal regions, and were associated with delayed peak in the right BA11 activation. We can hypothesize that communicating by examples (*Some…*) as opposed to general principles (*All…*) facilitate listeners’ general intelligence in both fluid [38], [39] and crystallized [40] counterparts. Being BA11 – presently observed as the crucial structure for Induction/Deduction processing, whose latency delay associates with Induction vs. Deduction delay– part of the full multiple-demand (MD [41]) system, we can conceive that exemplificative messages facilitate a systemic activation of the full cognitive network.

### Right parieto-frontal dominance in naturalistic Aristotelian contradiction identification

Induction/Deduction and contradiction-related contrasts revealed consistent right-hemisphere prevalence of frontal lobe involvement in both EEG and fMRI data.

The same regions in right prefrontal cortex and inferior parietal lobe (right MeFG/ACC, rBA 10/32) emerging from the Induction/Deduction contrast have been shown to be more active for reasoning than for calculation and correlate with reasoning load, whereas regions in left prefrontal cortex and superior parietal lobe were more active for calculation than for reasoning [11]. Compared to other studies that reported a preferential contribution of the left hemisphere for reasoning processes [5], [7], [8], we observed a clear right hemispheric dominance in identifying contradiction in naturalistic ASoO frame. We can only speculate on potential reasons for the apparent discrepancy, one of which may relate to the type of stimuli used. In this study, in contrast to formal symbolic reasoning, *All* and *Some* statements were applied to objects of everyday life (e.g. trees, books). These might have encouraged participants to use object imagery to solve the task. Processing of objects in working memory is typically associated with the right hemisphere activity [42]-[44]. An alternative explanation might in the belief conflict, inherent to the chosen task for this study. It has been observed that when a logical argument results in a belief conflict, reasoning is supported by right prefrontal cortex activation [3]. In our experiment, participants were exposed to some level of belief incongruence either when processing the premise or the conclusion, even though they were instructed to ignore the truthfulness of the statement. The construction of logically contradictory statements implies that one of them is to some extent in conflict with experience-based belief. For example a statement that ‘*All* books are thick’ is in conflict with our prior knowledge that ‘*Some* books are thin’. The stronger activation while processing a universal rather than a particular conclusion seems to support this hypothesis of a belief conflict, as general statements are invariably more likely to induce a belief conflict than particular ones (as in the example above or for another example ‘All exams are simple’ vs. ‘Some exams are simple’). Furthermore, particular and universal quantifiers largely require magnitude estimate and population size imagery which has been ascribed to the right prefrontal [45], [46] and intra-parietal cortices [47] providing common coding of number symbols and numeric coding, independent of dots, digits, and number words representations.

### Overall task relevant activations

The fMRI results of this study confirmed the well-established notion that reasoning is a highly complex cognitive process that relies on distributed cortical networks. These include the left precuneus and inferior parietal (bilateral angular) cortex, both associated with reading and processing of visual-verbal stimuli [48]; the bilateral prefrontal cortices (superior and medial frontal gyri), associated with executive functions, working memory and decision making processes [49]–[51]; premotor cortices (BA 6) bilaterally, associated with motor response and anterior cingulate cortices (BA 24,32), associated with learning and conflict resolution processes [52], [53].

In conclusion, the protocol chosen for the current EEG and fMRI reasoning study has some interesting properties in facilitating the identification of distinct cognitive processes involved in deciding whether two statements are contradictory. The perfect symmetry of the premise-conclusion logical structure used in this study has the advantage of being sensitive to isolate the process underlying the manipulation of the logical operators (*All/Some*) minimizing the linguistic and semantic confounds. Using this we have, for the first time, been able to identify the network involved in determining contradiction in logic. Investigating brain-behavior relationships we found that processing general statements is associated with longer processing time. Significant brain-behavior relationships strengthened the reliability of brain involvements in the contradictory judgment as assessed by the present study and honed the understanding of why specific cases are superior for human communication than general declarations.
